# State of the art of PET/MRI for rectal cancer: the added value to conventional imaging

**DOI:** 10.1590/0100-3984.2025.0060

**Published:** 2025-12-08

**Authors:** Poliana Fonseca Zampieri, Daniel Barros Garcia Hernandes, David Alberto Gutiérrez Albenda, Natally Horvat, Onofrio Antonio Catalano, Carlos Alberto Buchpiguel, Marcelo Araújo Queiroz

**Affiliations:** 1 Department of Radiology and Oncology, Hospital das Clínicas da Faculdade de Medicina da Universidade de São Paulo (HC-FMUSP), São Paulo, SP, Brazil; 2 Department of Radiology, Memorial Sloan Kettering Cancer Center, New York, NY, USA; 3 Department of Radiology, Massachusetts General Hospital, Harvard Medical School, Boston, MA, USA

**Keywords:** Positron-emission tomography, Magnetic resonance imaging, Rectal neoplasms, Tomografia por emissão de pósitrons, Ressonância magnética, Neoplasias retais.

## Abstract

Imaging plays a critical role in the assessment of patients with rectal cancer, and positron emission tomography/magnetic resonance imaging (PET/MRI) has shown superiority in specific clinical scenarios. This review describes the potential contribution of ^18^F-fluorodeoxyglucose (^18^F-FDG) PET/MRI relative to standard of care imaging-computed tomography (CT), MRI, or PET/CT-in the evaluation of patients with rectal cancer in settings such as primary staging, treatment response assessment, and recurrence detection. We discuss ^18^F-FDG PET/MRI protocols and clinical workflow, as well as highlighting the potential clinical superiority of PET/MRI over other imaging modalities.

## Abstract (pt)

Os exames de imagem desempenham um papel fundamental na avaliação de pacientes com câncer retal, e a tomografia por emissão de pósitrons/ressonância magnética (PET/RM) tem demonstrado superioridade em cenários clínicos específicos. Esta revisão descreve a potencial contribuição da PET/RM com ^18^F-fluorodesoxiglicose (^18^F-FDG) em relação à imagem padrão - tomografia computadorizada (TC), RM ou PET/TC - na avaliação de pacientes com câncer retal em cenários como estadiamento primário, avaliação da resposta terapêutica e detecção de recidiva. Discutimos os protocolos e o fluxo de trabalho clínico da PET/RM com ^18^F-FDG, além de destacar a potencial superioridade clínica da PET/RM sobre outras modalidades de imagem.

## INTRODUCTION

Colorectal cancer, in many statistical reports, includes malignancies of the colon, rectum, and anus^**(^1^)**^. It is currently the third most commonly diagnosed cancer and the second leading cause of cancer-related deaths among adults in the United States. The lifetime risk of developing colorectal cancer is approximately 4%, with 53,000 persons expected to have died from the disease in 2024^**(^2^,^3^)**^. This type of cancer accounts for 9.6% of all newly diagnosed cases and 9.3% of all cancer-related deaths, with higher incidence and mortality rates in countries with a high human development index^**(^1^)**^.

Hybrid positron emission tomography/magnetic resonance imaging (PET/MRI) systems acquire anatomical and metabolic data in a single examination, much like positron emission tomography/computed tomography (PET/CT) systems^**(^4^,^5^)**^. However, PET/MRI provides several advantages over PET/CT. Those advantages include simultaneous data acquisition for better image alignment, 20-60% less radiation exposure, enhanced soft-tissue contrast, and the ability to correlate multiparametric MRI and PET data. That allows the *in vivo* investigation of the biological features of cancers and tumor heterogeneity, among other advantages^**(^6^-^11^)**^. Despite its advantages, PET/MRI has certain limitations in comparison with PET/CT, including inferior performance in detecting lung nodules smaller than 7 mm, longer acquisition times, higher equipment costs, and a lack of standardized acquisition protocols for interinstitutional reproducibility^**(^5^,^9^)**^.

The role of PET/MRI in rectal cancer is still not fully defined. The literature suggests the utility and superiority of this technology in certain clinical scenarios when compared with conventional or hybrid imaging modalities (CT, MRI, or PET/CT). These include restaging after chemoradiotherapy (CRT), identifying local recurrence, managing treated patients with oligometastatic disease, and selecting patients who could benefit from rectum-sparing approaches^**(^10^,^12^,^13^)**^.

The radioactive glucose analogue ^18^F-fluorodeoxyglucose (^18^F-FDG) is injected intravenously and accumulates in areas of high glucose metabolism^**(^14^)**^. This work discusses the ^18^F-FDG PET/MRI protocol requirements for rectal cancer, aiming to establish a clear clinical workflow. The objective is to highlight the role of PET/MRI in various clinical scenarios of rectal cancer in comparison with conventional imaging and to demonstrate its potential clinical superiority over other imaging modalities.

## TECHNICAL ASPECTS

### Hybrid PET/MRI protocol

#### Technical requirements

Most of the technological challenges that hampered the development of PET/MRI systems (e.g., magnetic field inhomogeneities induced by ferromagnetic PET components, loss of PET data counts caused by MRI radiofrequencies, and deflection of electron paths in classical photomultiplier tubes due to the static MRI magnetic field) have been overcome, leading to the development of current hybrid integrated PET/MRI systems^**(^15^,^16^)**^. Similarly, combined approaches that consider atlas-based models, Dixon-based tissue decomposition, and artificial intelligence solutions have overcome the vast majority of the problems previously faced by PET/MRI in estimating attenuation correction (AC); that is, better estimation of AC in bones or in the case of metallic implants and continuous AC coefficients. In addition, using MRI instead of CT data results in a loss of AC accuracy in PET data^**(^17^)**^. Furthermore, MRI-based AC (MRAC) is based on a tissue classification using the T1-weighted Dixon MRI sequence rather than relying on the tissue density used for PET/CT AC (CTAC). Post-processed Dixon imaging generates four distinct sequences: water-only, fat-only, in-phase, and out-of-phase. By integrating this tissue information, an algorithm classifies the tissues as air, lung, fat, or soft tissue^**(^18^)**^.

It has been shown that MRAC ignores bones, assumes uniform attenuation coefficients in the lungs, and experiences signal truncation in the arms due to the fact that the transaxial field of view (FOV) of MRI is relatively small compared with that of PET^**(^15^)**^. In addition, metallic implants may significantly compromise the diagnostic accuracy and AC of PET/MRI scans if not properly managed^**(^16^)**^.

Building optimized imaging protocols to reach the highest diagnostic accuracy in a limited time is another challenge^**(^15^,^16^)**^. The mean scan time with dedicated protocols should vary in the range of 20-60 min^**(^15^)**^. This could improve patient comfort and productivity^**(^12^)**^, making the technology more cost-effective.

### PET/MRI protocols

#### Clinical workflow

As demonstrated in previous studies^**(^19^,^20^)**^, patient preparation for PET/MRI is important to minimize tracer uptake in normal tissues (kidneys, bladder, skeletal muscle, myocardium, and brown fat) while ensuring the maintenance and optimization of tracer uptake in the target structures (tumor tissues). For the PET portion, the preparation is the same as that for PET/CT^**(^19^)**^. For PET/MRI, contraindications (e.g., metallic inclusions, pregnancy, claustrophobia, passive implants, and active implants) should be identified^**(^11^)**^.

Typically, ^18^F-FDG is administered at a dose of 4.5 MBq/kg^**(^19^)**^. After ^18^F-FDG injection, the patient rests for 20-40 min and is then transferred to the PET/MRI scanner for positioning. To minimize bowel motion, scopolamine butylbromide or glucagon can be injected immediately before the investigation starts, a practice that is used as a clinical standard in many institutions^**(^11^)**^.

The region of the body to be scanned is divided into smaller sections called “beds”, which correspond to the size of the PET detector ring. The axial length of each bed is 25 cm, with bed positions overlapping by 23%. Depending on the height of the patient, 3-5 beds are usually needed for a whole-body (head-to-thigh) study^**(^16^)**^.

**Figure 1:**
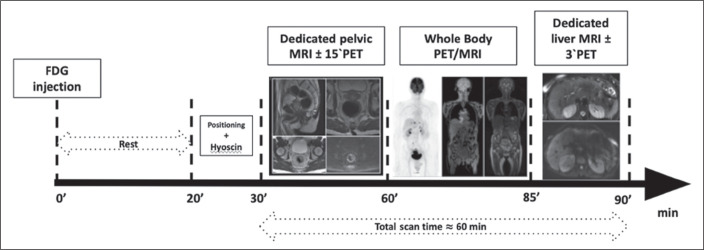
Clinical workflow in ^18^F-FDG PET/MRI for rectal cancer staging.

As summarized in Figure 1, the ^18^F-FDG PET/MRI scanning comprises three parts^**(^21^)**^: dedicated pelvic MRI (with 15-minute PET to increase sensitivity), which is carried out for locoregional staging of primary rectal cancer and follows the guidelines of the European Society of Gastroenterology and Abdominal Radiology; whole-body PET/MRI, which uses a 3-5 min/bed position acquisition time under three-dimensional (3D) image acquisition and standard reconstruction protocols; and dedicated abbreviated (3-10 min) liver MRI, with or without PET acquisition.

#### Dedicated pelvic MRI

The rectal protocol should include at least two-dimensional T2-weighted fast spin-echo sequences in the sagittal, coronal oblique, and axial oblique planes, with a slice thickness ≤ 3 mm, as well as a diffusion-weighted imaging (DWI) sequence (including at least one acquisition with a b-value ≥ 800). Adequate angulation to the axis of the rectal tumor should be used in the transverse (perpendicular) and coronal (parallel) sequences to avoid volume averaging. When assessing distal tumors, it is important to include a coronal sequence that is angled parallel to the anal canal to evaluate the relationship between the tumor and the anal sphincter. Fat-suppressed T1-weighted sequences, whether unenhanced or contrast-enhanced, are not typically recommended; nor are dynamic contrast-enhanced sequences^**(^22^)**^.

To improve the accuracy of ^18^F-FDG PET/MRI in detecting hypermetabolic lymph nodes (LNs), Bailey et al.^**(^23^)**^ performed a 15-min extended PET acquisition in the pelvis (simultaneously with the acquisition of the dedicated pelvic MRI). This resulted in detection of 40% more ^18^F-FDG-avid LNs (compared with the number detected with the standard 3-min PET acquisition), as well as LN upstaging in more than half of the patients. The authors explained that the increased detection of LNs with longer PET acquisition time was likely due to improved emission counts for each voxel. This may have improved the signal-to-noise ratio and made it easier for the radiologist to identify abnormal LNs. However, this approach may compromise the specificity of the study.

Recently, PET/MRI has been shown to enhance the evaluation of peritoneal metastases and outperform all standard of care imaging (SCI) modalities. Although the sensitivity of PET/MRI (97%) was higher than that of SCI (54%), the two were comparable in terms of their specificity (95% and 98%, respectively). In addition, PET/MRI findings, which were consistent with peritoneal carcinomatosis, were not detected on SCI and led to changes in treatment^**(^24^)**^.

#### Whole-body PET/MRI

Current whole-body PET/MRI protocols generally consist of a whole-body and a dedicated rectal acquisition^**(^20^)**^. The study starts with the acquisition of MRI localizer images (the equivalent of a CT scout scan in a PET/CT examination) to define the axial range primarily for the AC^**(^15^,^20^)**^. The T1-weighted Dixon sequences, despite the short acquisition time, can be used not only for AC but also for anatomic allocation of PET-positive lesions, with efficacy comparable to that of low-dose CT^**(^25^)**^. In addition, DWI can be incorporated into the standardized whole-body protocol during abdominal cavity scans, to enhance the detection of peritoneal carcinomatosis^**(^11^)**^.

After the images have been acquired for the MRAC, various pulse sequences can be used for specific bed positions or the whole-body overview, whereas PET data are acquired simultaneously. Depending on the specific clinical indication, the basic choice of the examination is adapted to each body compartment^**(^15^,^16^)**^. Altogether, the whole-body PET/MRI part of the examination usually takes 20-30 min. Although organ-dedicated PET/MRI acquisition can be obtained with an independent MRI approach, they are often acquired simultaneously with PET^**(^20^)**^.

#### Dedicated liver PET/MRI

In patients with rectal cancer, the liver is the most common site for distant metastasis. More than 50% of all patients experience liver metastasis during the course of the disease^**(^20^,^23^)**^.

The synergy among the picomolar sensitivity of PET, superior anatomic layout of MRI, higher contrast-to-noise ratio of dynamic contrast-enhanced MRI, and detection capability of DWI translates into improved performance of PET/MRI over stand-alone MRI for sensitivity (95% vs. 88%), specificity (97% vs. 98%), positive predictive value (97% vs. 98%), and negative predictive value (95% vs. 90%). In one dedicated study^**(^26^)**^, the area under the curve was found to differ between PET/MRI and MRI (95% vs. 92%). In addition, PET/MRI has been shown to characterize the vast majority of lesions considered indeterminate on MRI alone^**(^10^,^16^,^26^)**^. The performance of gadoxetic acid-enhanced liver MRI has been shown to be superior to that of CT and PET/CT for detecting and characterizing liver lesions, although the differences in comparison with PET/MRI were not significant^**(^26^)**^. Furthermore, PET can help detect concomitant extrahepatic metastases^**(^20^)**^. Contrast-enhanced MRI of the liver should be acquired. That examination should include T2-weighted and DWI sequences with 3D factors using navigators for respiratory gating, as well as T1-weighted 3D fat saturated (breath-hold) sequences, acquired before and after standard intravenous contrast or hepatobiliary specific contrast-during the arterial and portal phase, as well as at 4 min and 10 min after the injection of contrast^**(^11^)**^.

## CLINICAL INDICATIONS

The diagnosis of rectal cancer is based on the patient history, physical examination, and the serum level of carcinoembryonic antigen (CEA), together with digital rectal examination and endoscopy with biopsy for histopathological confirmation^**(^27^)**^. Patients with rectal neoplasms suitable for resection need a complete staging evaluation, which includes rigid proctoscopy and total colonoscopy to check for synchronous lesions and other pathological conditions in the colon and rectum^**(^27^)**^.

Pelvic MRI is recommended to define locoregional clinical staging (T and N stages) and predict the risks of local recurrence as well as synchronous or metachronous distant metastases by identifying extramural vascular invasion and distance to the mesorectal fascia. To define the presence of metastases (M stage), contrast-enhanced CT of the thorax and abdomen is recommended ^**(^27^)**^. If liver-directed therapy or surgery is being considered, a liver MRI with intravenous extracellular or hepatobiliary gadolinium-based contrast is preferred over CT to accurately assess the number and distribution of metastatic lesions^**(^27^)**^. Studies have shown that PET/CT can provide additional information to characterize indeterminate lesions on contrast-enhanced CT, evaluate potentially curable metastatic disease, and stage patients at high risk of metastases, particularly those with extensive extramural vascular invasion or elevated CEA levels^**(^21^,^27^)**^.

### Primary staging

The prognosis for patients with rectal cancer relies heavily on the stage of the disease at diagnosis. The earlier the cancer is identified, the higher is the likelihood of survival for at least five years after diagnosis. The overall five-year survival rate differs significantly, from 90% for localized tumors to just 18% for cases with metastatic disease^**(^28^)**^. In this context, the use of PET/MRI for staging primary rectal cancer integrates the standard imaging techniques with PET for assessing the T and N stages, as well as employing PET plus liver MRI for the M stage^**(^21^)**^. In addition, PET/MRI could replace all of these modalities for rectal cancer staging, offering valuable information in a single location and reducing the number of unnecessary treatments.

Because of its high resolution for soft tissues and reliable assessments of both tumor stage and the distance to the mesorectal fascia, which are necessary for appropriate surgical planning, MRI is the gold standard for T staging^**(^29^)**^. However, distinguishing between cancerous tissue and inflammatory or ischemic changes surrounding a tumor can be challenging^**(^30^)**^.

**Figure 2:**
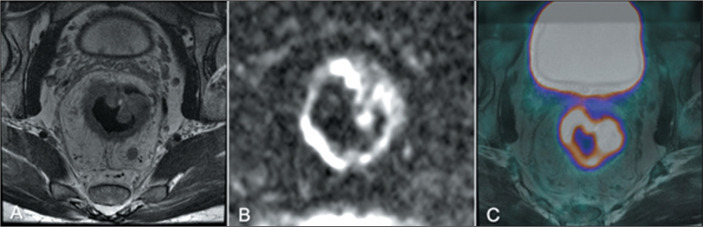
A 56-year-old male at primary staging of rectal cancer. The primary tumor and its extensions are better seen in the MRI component (A). DWI (B) aids in the detection of viable tumors but is not strictly necessary in primary staging. The PET component (C) shows a very high ^18^F-FDG uptake, and PET parameters can be quantified, helping identify high-risk patients .

The combination of ^18^F-FDG PET and MRI facilitates image co-registration and localization of metabolic events corresponding to morphologic abnormalities^**(^30^)**^. In addition, PET/MRI can improve the confidence of image readers and assist in characterizing tumor extension beyond the muscularis propria layer^**(^29^)**^. The hypermetabolism of rectal cancer is highlighted relative to the non-hypermetabolic areas of ischemia, low-grade inflammation, and diverticulosis-induced thickening of the sigmoid wall, aiding in its differentiation^**(^30^)**^, as illustrated in Figure 2.

One significant advantage of ^18^F-FDG PET is its ability to quantitatively describe tumor metabolism using several biomarkers. In addition to the maximum standardized uptake value, the predictive roles of metabolic tumor volume and total lesion glycolysis in primary lesions may help select high-risk patients. One study correlated ^18^F-FDG PET/CT measurements and pathology of a tumor specimen in patients with rectal cancer, and higher metabolic tumor volume values was found to have a stronger correlation with pT3-pT4 staging. This information could be valuable for identifying patients who may benefit from preoperative CRT or more aggressive treatment options^**(^31^)**^. Regarding N staging, MRI alone can easily characterize nodules > 1 cm, but that specificity drops somewhat for nodules < 1 cm and even more for those < 5 mm. Hypermetabolism on PET appears to have a higher specificity for characterizing small nodules than do findings on MRI alone^**(^28^)**^. For N staging, Catalano et al.^**(^30^)**^ showed that PET/MRI was significantly superior to MRI alone, with specificities of 79% and 58%, respectively.

To enhance the accuracy of ^18^F-FDG PET/MRI in detecting hypermetabolic LNs, imaging protocols must be optimized^**(^23^)**^. Although longer PET acquisitions (corresponding to the time spent simultaneously acquiring MRI data) result in higher sensitivity for detecting small perirectal nodules, that also reduces the specificity^**(^29^)**^ and increases the possibility of falsely upstaging and overtreating patients^**(^23^)**^. However, correlating LN metabolic data with morphologic features (such as irregular or indistinct contours, internal heterogeneity, loss of the fatty hilum, and round shape) might improve overall PET/MRI accuracy^**(^30^)**^.

A study conducted during the initial staging of rectal cancer in 101 patients demonstrated that PET/MRI had a sensitivity of 90.8% and a specificity of 86.1%. In comparison, conventional staging methods, including pelvic MRI and thoracic and abdominal contrast-enhanced CT, achieved an accuracy of 82.6% for detecting distant metastases. Those findings indicate that, in comparison with conventional imaging methods, PET/MRI offers greater accuracy for identifying synchronous distant metastases in patients with extramural vascular invasion, as well as a higher detection rate for non-regional lymphadenopathy, liver lesions, and lung lesions. In addition, PET/MRI has been shown to facilitate the characterization of indeterminate lesions that were not clearly defined in conventional staging^**(^21^)**^.

### Characterization of the mucinous component

Mucinous rectal carcinoma, characterized by having over 50% extracellular mucin in its tumor composition, exhibits several genetic abnormalities and demonstrates greater aggressiveness and resistance to therapy compared with nonmucinous rectal adenocarcinomas. On MRI, mucinous rectal cancers show significantly higher T2-weighted signal intensity, less enhancement, and diffusion restriction than do nonmucinous tumors^**(^32^)**^. In fact, the mucinous components do not exhibit restricted diffusion^**(^33^)**^.

**Figure 3:**
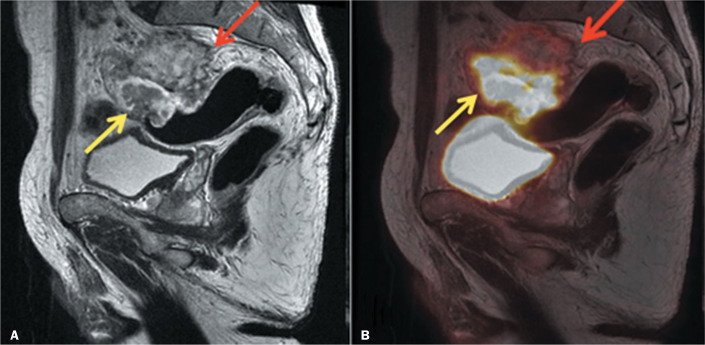
A 56-year-old male at primary staging of rectal cancer. A: MRI detects a lesion with an extensive mucinous component (red arrow) and some nonmucinous components (yellow arrow). B: PET/MRI clearly shows the difference between the mucinous and nonmucinous components by highlighting ^18^F-FDG avidity.

Depending on the degree of mucin and solid content, PET/CT and PET/MRI show variable ^18^F-FDG uptake^**(^34^)**^. Low or even a lack of ^18^F-FDG uptake by mucinous tumors (Figure 3) has been attributed to the relative hypocellularity of these malignancies, which may result in false-negative cases^**(^34^,^35^)**^. However, one study showed that the lower ^18^F-FDG uptake in mucinous colorectal cancers could derive from studies in which PET (and not PET/CT or PET/MRI) imaging was used, and the precise anatomical delineation of these tumors allowed an adequate estimation of ^18^F-FDG uptake. The authors suggested that the solid components of the tumors appeared to be extremely avid for ^18^F-FDG, possibly compensating for the low uptake of the mucinous component^**(^35^)**^. Because PET/MRI provides a more comprehensive evaluation of the primary rectal tumor, it allows better differentiation of tissue components within the same tumor^**(^36^)**^.

### Treatment response assessment

**Figure 4:**
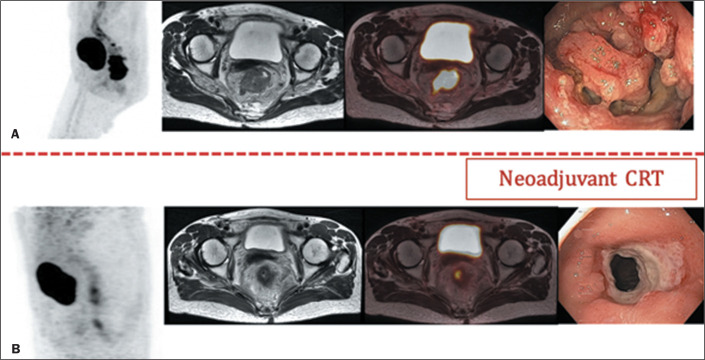
A 48-year-old female at primary staging (A) and treatment response assessment following neoadjuvant CRT (B). PET/MRI after neoadjuvant CRT showed a partial metabolic response with residual ^18^F-FDG uptake suggestive of a viable tumor. The patient underwent surgery that confirmed the staging as ypT3ypN0.

Although surgery plays a crucial role in the treatment of rectal cancer, surgical resection is typically reserved for patients with localized disease. For those with more advanced disease, neoadjuvant therapy is an essential part of the treatment plan. The aim of neoadjuvant therapy is to shrink the tumor, facilitate complete surgical resection, and lower the risk of local recurrence (Figure 4). After neoadjuvant CRT, a “watch-and-wait” approach has emerged as a potential option for a select group of patients. In this strategy, individuals who show a complete clinical response to neoadjuvant therapy are closely monitored instead of proceeding directly to surgery. This approach helps avoid the complications associated with surgery^**(^37^)**^.

**Figure 5:**
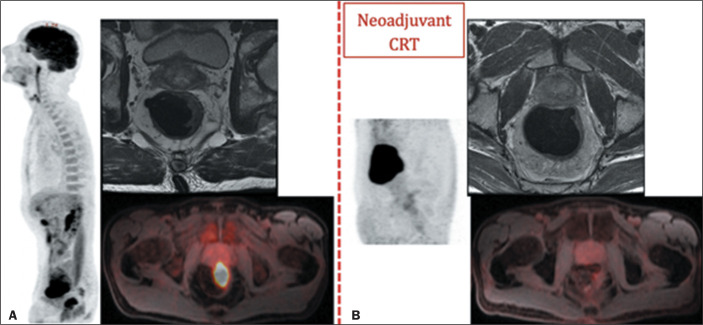
A 57-year-old male at primary staging (A) and treatment response assessment following neoadjuvant CRT (B). At primary staging, PET/MRI detected a hypermetabolic primary rectal tumor staged as T3N0 (A). PET/MRI after neoadjuvant CRT showed a complete metabolic response, although a partial morphology response (tumor regression grade 3) was depicted by MRI (B).

A meta-analysis investigating the use of PET/CT in the reassessment of locally advanced rectal cancer after CRT revealed that radiological PET/CT features correlated with histopathological evaluations of tumor regression^**(^38^)**^. Although MRI is commonly used for surgical planning after CRT, it has several limitations as a predictor of treatment response. Changes in tumor or nodule size do not correlate well with treatment response, and DWI is often limited by artifacts. Combining DWI with ^18^F-FDG PET may improve the characterization of the treatment response. In many cases, discrepancies can arise between changes in size or diffusion and changes in metabolism, suggesting a response different from that based on MRI alone^**(^29^)**^, as depicted in Figure 5.

### Detection of recurrence

Up to 40% of patients with rectal cancer experience local or distant recurrence, with the risk of local recurrence ranging from 4% to 10%^**(^39^)**^. In those with rising CEA levels and inconclusive CT results, PET is the most sensitive and specific method for detecting recurrence^**(^40^)**^. One study found that PET/CT is useful for detecting recurrence in patients with normal CEA values who also exhibit suspicious clinical or radiologic findings^**(^40^)**^.

**Figure 6:**
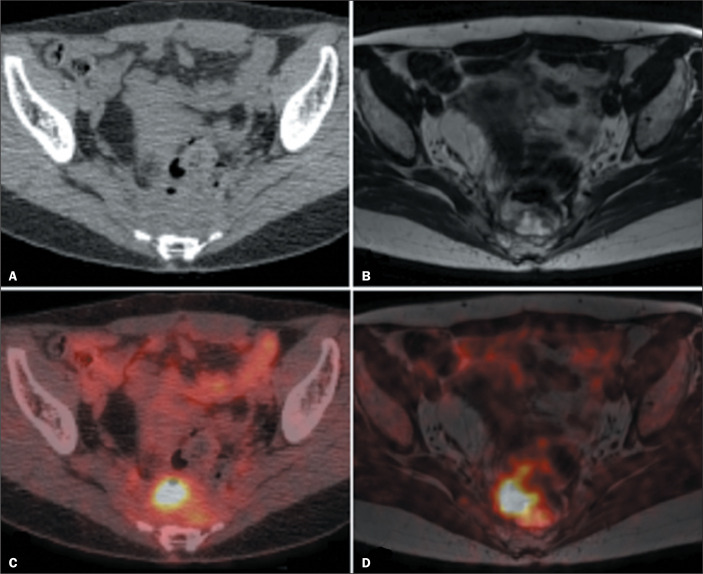
Restaging in a 57-year-old male with rectal cancer and rising CEA levels. PET/CT (B) depicts ^18^F-FDG uptake without a clear morphological change on CT (A). However, local recurrence was detected on MRI (C) and PET/MRI (D).

The use of ^18^F-FDG PET combined with MRI may assist in distinguishing between post-therapy scar or desmoplastic reaction and residual tumor or local recurrence^**(^29^)**^, as shown in Figure 6. Accurate knowledge of the invasion of adjacent structures, such as the piriform muscles, sacral bone, and lumbosacral nerves, is essential when planning surgical resection^**(^41^)**^. In cases of nonoperative treatment, characterization of the complete pathologic response is fundamental^**(^29^)**^. The hybrid imaging technique PET/MRI offers functional imaging with high sensitivity and specificity for detecting recurrence. It also provides excellent soft tissue contrast, facilitating the assessment of the extent of local and distant recurrence^**(^13^,^24^,^42^)**^.

## CLINICAL IMPACT OF PET/MRI FINDINGS

**Figure 7:**
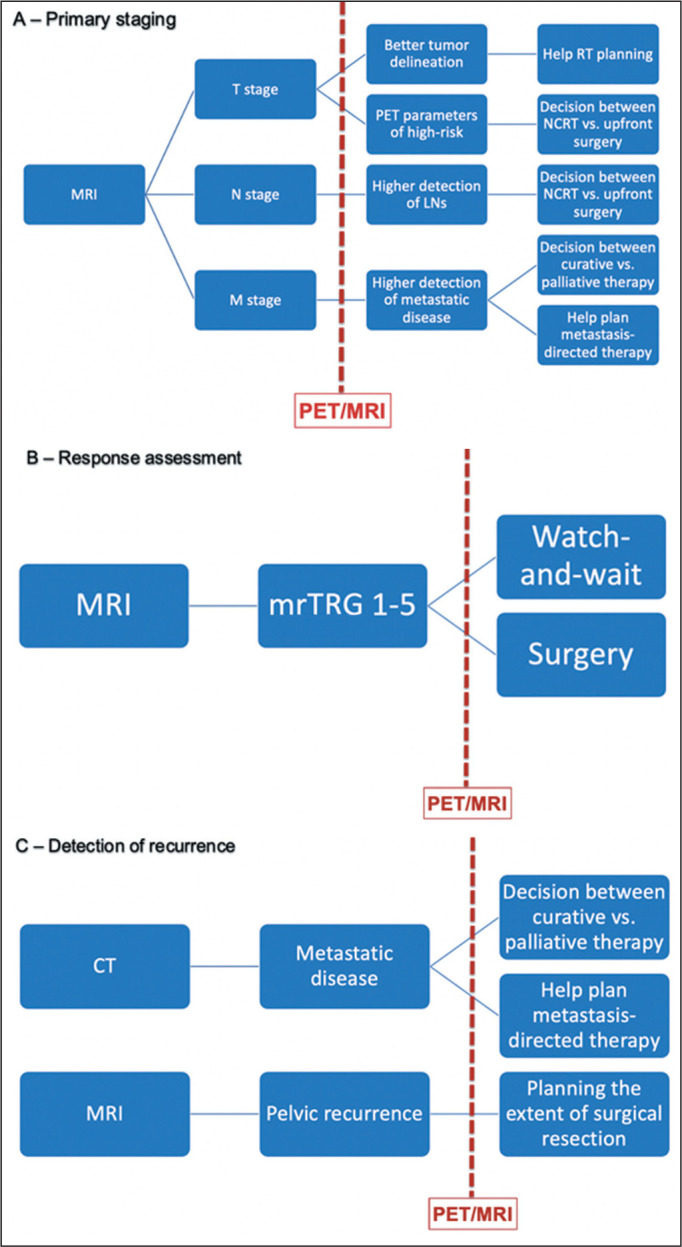
Clinical impact of PET/MRI on primary staging of rectal cancer (A), treatment response assessment (B), and detection of recurrence (C). nCRT, neoadjuvant CRT; RT, radiotherapy; mrTRG, magnetic resonance tumor regression grade.

Imaging is key for clinical decision-making in rectal cancer, defining the most appropriate therapeutic approach based on MRI characteristics and potentially additional findings from PET or CT^**(^27^)**^. In various clinical settings, PET/MRI can help characterize the tumor, nodal, and metastatic status of patients with rectal cancer (Figure 7).

In a primary staging setting, PET/MRI may be incremental to the tumor staging, providing better delineation of the primary tumor, especially regarding sphincter complex infiltration. This can not only facilitate the planning of the radiotherapy fields but also add semiquantitative parameters (such as the maximum standardized uptake value, metabolic tumor volume, and total lesion glycolysis) that can be used as prognostic biomarkers^**(^39^)**^. For nodal staging, PET/MRI provides a higher detection rate of suspicious LNs, which may be crucial in deciding between neoadjuvant CRT and upfront surgery^**(^23^,^43^)**^. For metastasis staging, the diagnostic accuracy of PET/MRI is higher than that of conventional staging for detecting synchronous metastases. This helps to plan metastasis-directed therapy, influencing the curative and palliative intentions of treatment^**(^21^,^44^)**^.

In the assessment of treatment response following neoadjuvant CRT, the metabolic behavior of the primary tumor, characterized by PET/MRI, facilitates the decision regarding organ preservation^**(^45^)**^. When the PET and MRI findings both indicate significant tumor regression, the “watch-and-wait” strategy becomes more reliable. However, discordant imaging findings may prompt a more aggressive approach.

When detecting tumor relapse, PET/MRI accurately identifies the sites of recurrence, enabling the best clinical decision regarding the next therapeutic option. If the recurrence is limited to the pelvic region, PET/MRI helps surgeons plan the extent of pelvic exenteration. In metastatic recurrence, the high detection rate of PET/MRI (higher than that of CT) helps physicians decide between a curative and palliative approach^**(^42^)**^. The potential advantages of PET/MRI suggest that it can produce better oncological results while being more cost-effective than conventional imaging.

## PERSPECTIVES

### Gallium-68-labeled fibroblast activation protein inhibitor

Gallium-68-labeled fibroblast activation protein inhibitor (^68^Ga-FAPI) is a promising radiotracer in the evaluation of gastrointestinal cancer. It has emerged as a tracer for PET tumor imaging, showing advantageous pharmacokinetics and biodistribution *in vivo*, as well as providing a clear delineation of primary tumors and their metastases^**(^46^)**^.

Fibroblast activation protein is overexpressed in cancer-associated fibroblasts, whereas its expression is minimal in normal tissues and organs. That makes it an excellent molecular target for the diagnosis and treatment of neoplasms^**(^47^)**^.

Studies comparing ^68^Ga-FAPI with ^18^F-FDG in patients with gastrointestinal cancer have shown superiority of ^68^Ga-FAPI regarding the localization of primary and metastatic foci. In addition, because of its lower background activity (especially in the abdomen and pelvis), ^68^Ga-FAPI is considered superior for detecting peritoneal and liver metastases^**(^46^,^47^)**^.

## CONCLUSION

Combining metabolic and morphological data, ^18^F-FDG PET/MRI may contribute to the evaluation of patients with rectal cancer in multiple scenarios-tumor and node staging; characterization of mucinous components; detection of distant metastasis; treatment response assessment; and detection of recurrence-more accurately than conventional imaging modalities alone or PET/CT. However, the identification of lung metastases is a main aspect of patient management and ^18^F-FDG PET/MRI has lower sensitivity than does PET/CT for the detection of some small pulmonary metastases. In addition, ^18^F-FDG PET/MRI presents longer acquisition times and higher equipment costs, thus increasing administrative complexity and creating greater logistical challenges, as well as requiring financial adjustments and differentiated technical training. One specific limitation is a lack of standardized acquisition protocols for interinstitutional reproducibility. Therefore, it is crucial to find optimized imaging protocols to achieve the highest diagnostic accuracy without submitting the patient to a lengthy examination. Other limitations of PET/MRI include several technical considerations required in the design and operation of the imaging systems. Specifically, modifications to conventional imaging systems that accommodate integration of the two modalities without image-degrading cross-talk require a deeper understanding before the technology can be widely adopted.

Although a standardized template for hybrid imaging reporting does not currently exist, an array of structured reporting systems is available for CT, MRI, and ultrasound. Numerous reporting and data systems have been developed for specific conditions (e.g., the Vesical Imaging Reporting and Data System). Although evidence suggests that such systems do not directly affect reporting quality or diagnostic accuracy, they have been shown to promote uniformity in imaging and reporting outputs. A primary benefit of these systems is the consistency in terminology they afford, which enhance the reliability of reports and facilitate a clearer understanding on the part of referring physicians. Given the diverse array of reporting systems for PET and MRI-often organized by specific diseases or therapies-offering a concise recommendation on a preferred system is challenging. Nevertheless, institutions should implement standardized reporting for both components in PET/MRI.

Evidence of the real benefit of using PET/MRI in rectal cancer is limited. Because of the lack of randomized clinical trials to determine the overall impact on patient outcome in terms of survival benefit, evaluating the long-term advantages and costs of PET/MRI, this modality is not currently used in therapeutic decision-making. Currently, there is a clear need to obtain a better level of scientific evidence in those aspects, with the aim of developing protocols for the standardized use of PET/MRI in rectal cancer.

## Data Availability

Not applicable.
